# Automated Vision-Based Detection of Cracks on Concrete Surfaces Using a Deep Learning Technique

**DOI:** 10.3390/s18103452

**Published:** 2018-10-14

**Authors:** Byunghyun Kim, Soojin Cho

**Affiliations:** Department of Civil Engineering, University of Seoul, Seoul 02504, Korea; shdik2002@uos.ac.kr

**Keywords:** crack, deep learning, convolutional neural networks, AlexNet, unmanned aerial vehicle

## Abstract

At present, a number of computer vision-based crack detection techniques have been developed to efficiently inspect and manage a large number of structures. However, these techniques have not replaced visual inspection, as they have been developed under near-ideal conditions and not in an on-site environment. This article proposes an automated detection technique for crack morphology on concrete surface under an on-site environment based on convolutional neural networks (CNNs). A well-known CNN, AlexNet is trained for crack detection with images scraped from the Internet. The training set is divided into five classes involving cracks, intact surfaces, two types of similar patterns of cracks, and plants. A comparative study evaluates the successfulness of the detailed surface categorization. A probability map is developed using a softmax layer value to add robustness to sliding window detection and a parametric study was carried out to determine its threshold. The applicability of the proposed method is evaluated on images taken from the field and real-time video frames taken using an unmanned aerial vehicle. The evaluation results confirm the high adoptability of the proposed method for crack inspection in an on-site environment.

## 1. Introduction

Civil infrastructures are aging in most of the industrialized countries, which is associated with significant social issues. In the case of the United States, the condition of their infrastructure is graded as D+ on average, and the rehabilitation cost for bridges in the USA is expected to be $123 billion [1]. In Korea, due to a slightly later development from the 1970s, the percentage of structures older than 30 years was estimated as 3.8% in 2014, while it is expected to increase exponentially and reach 13.8% in 2024 and 33.7% in 2029 [2]. Similarly, most of the industrialized countries are paying attention to the development of cost-effective structural maintenance strategies using state-of-the-art information and communication technologies.

The vision-based technique, which uses imaging devices as sensors, is now emerging as the most effective tool for structural inspection and monitoring. Current advances made in the field of vision-based inspection and monitoring include noncontact deflection measurement [3,4,5], steel corrosion detection [6,7,8], and spalling detection [9,10]. In the last few decades, especially many attempts have been made to measure concrete cracks using the image binarization method [11], the stereo-vision method [12], and sequential image processing [13]. Abdel-Qader et al. (2003) [14] compared the effectiveness of four crack detection techniques: fast Haar transform, fast Fourier transform, Sobel edge detection, and Canny edge detection. Rabah et al. (2013) [15] implemented terrestrial laser scanning to detect cracks and suggested a three-step method composed of shading correction, crack detection, and crack mapping. Prasanna et al. (2016) [16] adopted a robotic imaging system and developed an automated crack detection algorithm called STRUM (spatially tuned robust multi-feature) classifier to detect cracks on bridge surfaces and succeeded in achieving a 95% performance accuracy.

But the vision-based technique has some limitations to be applied in the real world, since it is not easy to develop an algorithm that is able to cover all of the unexpected situations of the real world. Last several years, deep learning has been spotlighted as one of the most promising solutions for this problem. Deep learning refers to machine learning techniques based on artificial neural networks with many hidden layers for enhanced performance. It has shown outstanding performances especially in object detection [17,18,19], natural language processing [20,21,22], advertising [23], biology [24,25], and so on. Deep learning has been employed not only for the fields mentioned above but also for other engineering problems. Zhao et al. (2017) [26] combined deep learning with speeded-up robust features (SURF)-based approach to develop a traffic surveillance system which processes aerial imagery to track vehicles and their movements.

Of special note, there have been several efforts to advance crack measurement using deep learning techniques [27,28,29]. Tong et al. (2017) [29] proposed a two-step approach for pavement crack detection: preliminary selection of images possibly containing cracks using a k-means clustering analysis, and an application of a convolutional neural network (CNN) for training and testing using selected images. So far, the crack measurement techniques reports have been validated for near-ideal laboratory conditions as well as in the field. However, it was found that the test images reported in the literature contain cracks and intact surfaces only, though the real structures have various apparent conditions over cracks and intact surfaces. Hence, the testing under human-made conditions in the literature still has limitations in dealing with all real outdoor conditions that most of the structures are exposed to.

In this paper, an automated vision-based crack detection method using deep learning is proposed to pick out crack parts among a large dataset of images recorded under field conditions. One of the key contributions of this paper is the development of multiple classes including non-crack objects using training data collected from the Internet, which make the trained network capable to cover diversity of on-field environment. This method aims to facilitate the regular inspection of concrete structures and speed up the assessment of detailed crack distribution without losing accuracy using various cameras and vision devices, such as drones. The proposed method is composed of three steps: (1) collection of a large volume of images from the Internet with subsequent categorization into five classes (intact surfaces, cracks, multiple joints and edges, single joint or edge, etc.); (2) development of a deep CNN model using collected images and their augmentation; and (3) automatic selection of crack parts from test images using the trained deep learning model.

Contributions to the abovementioned literature are made in each step. In the first step, the Internet-based collection improves the collection of images taken under diverse structural, environmental, and photographing conditions and enables easy classification of collected images based on search keywords. In the second step, a transfer learning approach has been introduced to save time and cost involved in developing a deep learning model. In the third step, the probability map is introduced based on the last softmax output and overlapped searching to make the searching process robust. The whole procedure of the proposed method has been validated for the images of a building captured using a camera and a video of a concrete retaining wall recorded using a drone.

## 2. Methodology

### 2.1. Overall Framework of the Proposed Method

**Figure 1:**
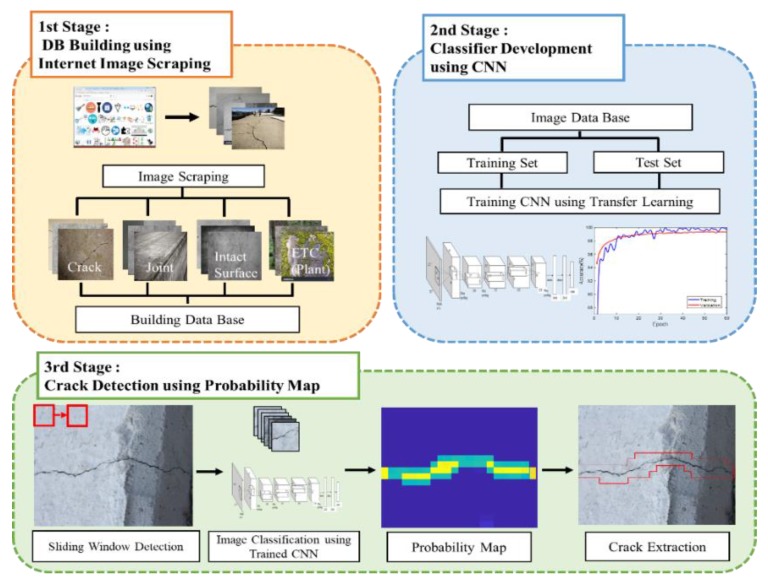
Overall framework of the proposed method.

Figure 1 illustrates the overall framework of the proposed crack detection method in this study. The framework is composed of three stages: (1) database (DB) building; (2) classifier development; and (3) crack detection. In the DB building stage, thousands of images are scraped from the Internet to create an image DB for deep learning model training. The images are scraped for various classes, such as cracks, intact concrete surfaces, and non-crack objects that can be easily misclassified as cracks. In the classifier development stage, a CNN classifier is developed to detect cracks against intact surfaces and non-crack objects. A transfer learning (i.e., fine-tuning of the developed neural network model) of the well-known AlexNet [30] is used to develop the classifier in this study. In the crack detection stage, the trained classifier scans a set of images from the testing structures by sliding a window whose size equals the input of the classifier. With overlapped scanning, a probability map of the classification is obtained from the output of the classifier. Given a probability threshold, groups of pixels whose probability exceeds the threshold are selected as possible crack parts. The details of three stages are described in the following sections.

### 2.2. First Stage: DB Building Using Internet Image Scraping

When training a classifier, the variety of the training images determines the performance of the classification. A CNN classifier trained with images under constrained conditions may display poor performance when classifying an image outside the considered conditions. Since obtaining training images under all possible conditions is very difficult, big data from the Internet may be the best source to obtain images taken under a wide variety of conditions.

**Figure 2:**
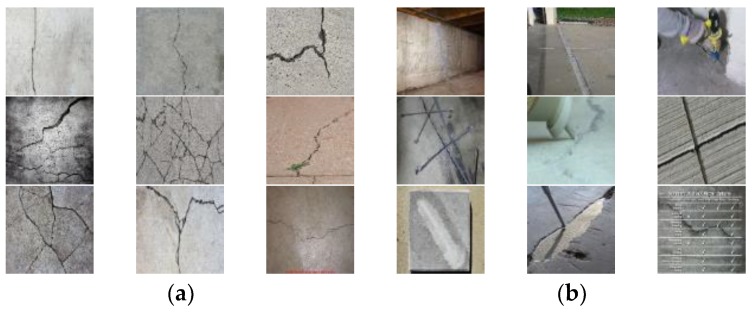
Examples of (**a**) valid and (**b**) invalid images scraped by the keyword “concrete crack”.

The image DB in this study was established using a commercial scraper, called ScrapeBox [31], which scrapes images from a search engine site (e.g., Google) for a keyword. The use of a scraper is beneficial in two aspects: (1) it can collect various types of images from a huge database on the Internet, and (2) it naturally provides images classified by the search keyword. Figure 2 shows examples of valid and invalid images scraped by the keyword “concrete crack”. The valid images contain single or multiple cracks on concrete or mortar surfaces, while the invalid images contain invisible, repaired, man-made cracks or irrelevant objects such as texts and company logos.

The number of scraped images and their validity checked by the manual pick-out process are tabulated in Table 1. For example, 497 images scraped by the keyword “concrete crack” were found to be valid for the training by manual pick-out from 723 images. Search with various keywords in different languages and subsequent manual pick-out resulted in 2073 valid crack images. In a similar manner, more than 1000 images were obtained for joints, corners, and plants, as shown in Table 1.

### 2.3. Second Stage: Classifier Development Using a CNN

The development of a deep learning model for crack detection is the key part of this research, but training a model from scratch takes a considerable amount of time even with a workstation-level computer or computers with parallel CPUs. Training of the AlexNet [30], which is a well-known deep CNN classifier, took five to six days on two NVIDIA GTX 580 3GB GPUs because of the large size of the training image set (150,000 images). The long training time prevents quick validation of the trained classifier with various training options.

**Figure 3:**
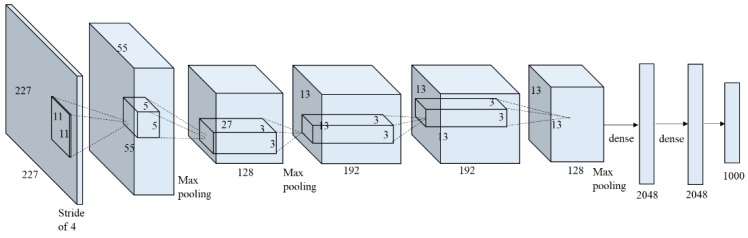
Overall architecture of AlexNet (redrawn from [30]).

Transfer learning reduces training time by fine-tuning a deep learning model that has been trained for a similar purpose. By starting the training on the pretrained model, and not on the randomly initialized model, the training process can be minimized. In this study, a CNN classifier for crack detection was developed using transfer learning of a CNN classifier developed for object detection, namely, AlexNet [30]. Since AlexNet [30] aims to classify objects in the images, it is a good model for transfer learning to classify cracks as objects in an image. AlexNet [30] consists of five convolutional layers followed by max-polling layers, and three fully-connected layers with a 1000-way softmax output as shown in Figure 3. AlexNet [30] implemented rectified nonlinear unit (ReLU) nonlinearity as an activation function at the end of neurons (except the final layer) to reduce the vanishing gradient effect. More details about AlexNet can be found in [30].

The MATLAB Neural Network Toolbox provides easy implementation of AlexNet [30] for transfer learning to develop an image classifier. In this study, the final layer of AlexNet [30], was changed to have five outputs to detect five different classes, namely Crack, Joint/Edge (Multiple Lines, ML), Joint/Edge (Single Line, SL), Intact Surface, and Plant. Then, the pretrained AlexNet [30] model was retrained using the image DB categorized into five classes.

The five classes are determined to minimize false detection of cracks. In real concrete structures, there are cracks as well as noncrack objects with thin and long shapes on the surface, e.g., joints, and sediments flowing down. Since a deep learning model automatically finds features representing each class during the training, a class containing various images without apparent similarity may result in poor feature representation of the class. Assuming that two classes (e.g., Crack class and Noncrack class) are used in the training in this study, the noncrack objects with thin and long shapes should be included in the Noncrack class with the other noncrack objects with different shapes (e.g., intact surface, wide pollution). Then, the thin and long shapes of the objects may be ignored in the representing features of the Noncrack class, while the shapes are representing the Crack class. Thus, the classifier misclassifies them into cracks due to shape similarity, as will be shown in Section 4.1.

**Figure 4:**
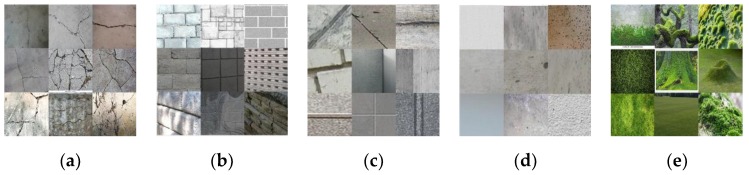
Examples of detailed categorization of images for crack detection: (**a**) Crack; (**b**) Joint/Edge (ML); (**c**) Joint/Edge (SL); (**d**) Intact Surface; and (**e**) Plant.

Image examples of the five classes are displayed in Figure 4. The images of various types of concrete cracks from macro to microcracks are categorized into Crack class (Figure 4a). The images containing multiple construction joints and joints between concrete tiles are categorized into Joint/Edge (ML) class (Figure 4b), and those that have one line or two lines at most into the Joint/Edge (SL) class (Figure 4c). To cover various types of real-world concrete surfaces, concrete surface images involving diverse texture and different colors are categorized into the Intact Surface class (Figure 4d). Finally, the images of moss-like plants which can be found on concrete surfaces are categorized into the Plant class (Figure 4e). This detailed categorization of images contributes to the high accuracy of the trained CNN model in detecting concrete cracks.

### 2.4. Third Stage: Crack Detection Using a Probability Map

The trained CNN classifier, has softmax outputs in the final layer, and the outputs correspond to the possibility of each class. The softmax function reduces down a real-valued *N*-dimensional vector *x* to an N-dimensional real-valued vector *σ*(*x*) in the range (0, 1) that add up to 1. The function is given as:

(1) $$\begin{array}{l} \sigma :{\mathbb{R}}^{N}\to {\left(0,1\right)}^{N}\\ \sigma {\left(x\right)}_{i}=\frac{e^{x_{i}}}{\sum_{k=1}^{N}e^{x_{k}}}\;\mathrm{for}\;i=1,\cdots ,N \end{array}$$

**Figure 5:**
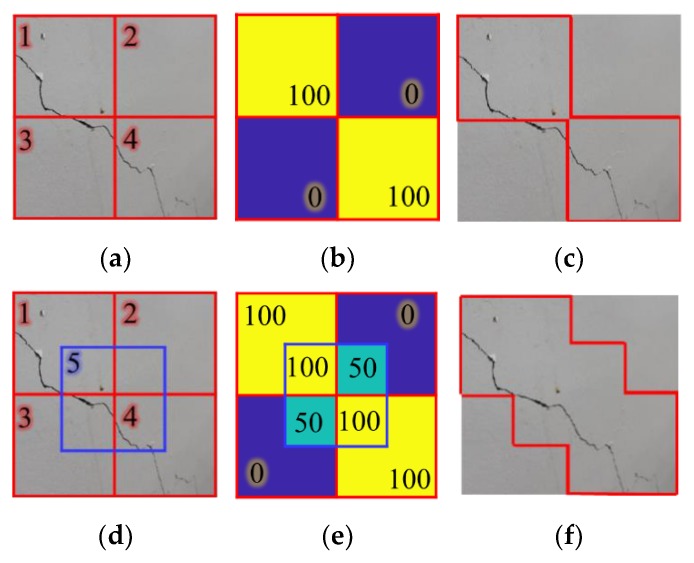
Performance enhancement using overlapped windows: (**a**) sliding windows without overlapping; (**b**) softmax output of the windows; (**c**) detection result from sliding windows without overlapping; (**d**) sliding windows with overlapping; (**e**) average softmax output of the windows; and (**f**) enhanced detection result with sliding windows with overlapping and probability map.

A CNN classifier, including AlexNet [30], has great but imperfect classification performance because of its fixed input layer size. Most of the images contain cracks around the corner of the image. Thus, the cracks located near the window border may be missing or misclassified during scanning using the trained classifier. Figure 5 illustrates this issue. If a window slides from 1 to 4 to scan the image of Figure 5a, the crack part located in the corner of Window 4 will be disregarded as shown in Figure 5b. Figure 5c shows that the crack detection result without overlapped windows has low accuracy on a pixel-level.

In this study, a probability map with an overlapped window sliding strategy is developed to overcome this issue. The test image is scanned using an overlapped window, and the probability map is obtained using the average softmax layer value of Crack class scanned by a sliding window with overlapping. Using this strategy, a crack object near the border of a window is located near the center of an overlapped window. Figure 5d shows an example of the addition of a 50% overlapped window to Figure 5a, and Figure 5e shows that the probability of the disregarded part of the crack in the detection result without overlapped windows was increased from 0% to 50%. By highlighting the pixels whose probability exceeds a predetermined threshold, 50% in this case, crack parts in the image can be extracted. The extracted crack parts in Figure 5f are more valid compared to the result of Figure 5c. The window sliding strategy may be changed according to the image size, allowable computing time, and target accuracy. Note that a window scans a test image twice with a quarter overlapping of the image as shown in Figure 5e in this study.

## 3. Development of a CNN Classifier

### 3.1. Data Augmentation

Since AlexNet has 60 million parameters to be trained, a number of training images must be prepared. Though a few thousand images were scraped from the Internet as shown in Table 1, they may not fully cover diverse photographing environments that significantly affect the accuracy of the trained classifier in practice. To overcome this hurdle, data augmentation is the most effective method for building up a number of training images by simulating diverse photographing environments, and reducing possible overfitting of the CNN classifier.

**Figure 6:**
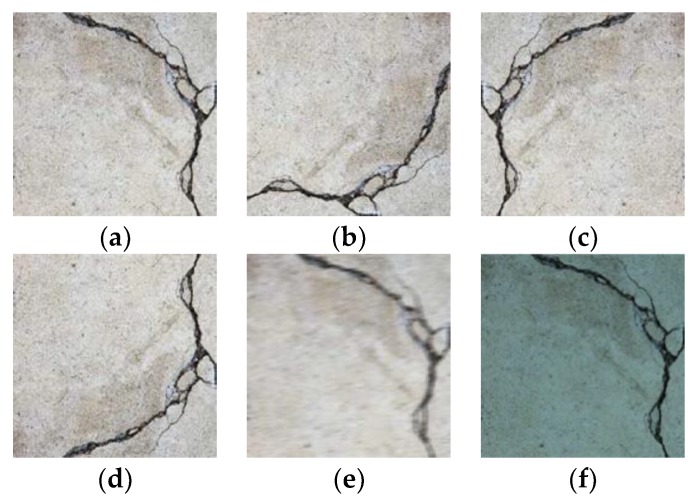
Example of image augmentation: (**a**) Original image; (**b**) rotation 90° to clockwise direction; (**c**) flip left to right; (**d**) flip up and down; (**e**) blur; and (**f**) color conversion.

The training images can be augmented in three ways: geometry transformation, blurring, and color conversion. Geometry transformation, which aliases translation, reflection, and rotation, considers the variation of direction and angle at which the images are taken. Blurring considers the possible instability of the imaging camera under insufficient light and unfocused shot. Color conversion, which aliases illumination of the color field (RGB), considers the variation of light and color characteristics of the imaging camera. Figure 6 shows examples of the image modification for data augmentation. Using data augmentation, the number of training data can increase up to at least ten times.

### 3.2. Training: Transfer Learning

**Figure 7:**
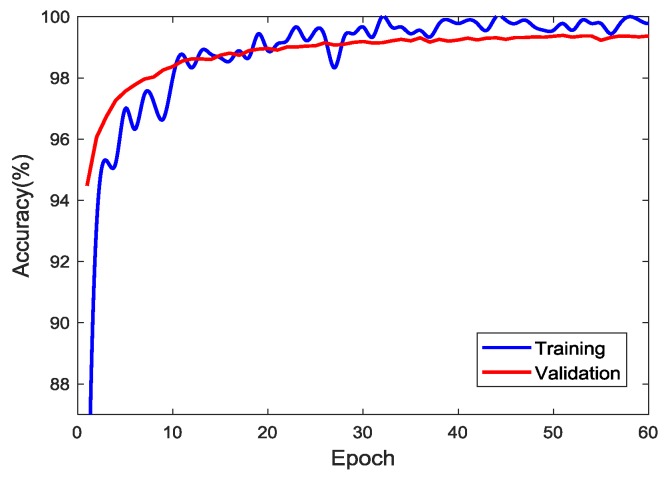
Accuracy of training and validation.

In this study, a personal computer (PC) with a single GPU (CPU: Intel(R) Core(TM) i3-6100, RAM: 8192 MB, GPU: NVIDIA Geforce 1060 3 GB) was used for the training with the help of transfer learning. The number of training images was increased to 10,000 for each class (i.e., a total of 50,000) by taking image augmentation techniques. Figure 7 shows the accuracy of training and validation as epochs proceed. To update the parameters using a stochastic gradient descent algorithm, the network takes a subset of the image data set, called a “mini-batch”, for each iteration. Once the network completes its pass through the full training set, it completes one epoch. The training accuracy is calculated based on 40,000 images, and validation based on 10,000 images not included in the training set. The highest accuracies in the training are 100.00% at several epochs and 99.39% at the 51st epoch in the validation. Despite the computer with relatively low computational power, the training during 60 epochs took 316 min since the training started from the parameters of the pretrained AlexNet. The result that the accuracy of validation reached 98% at 8th epoch shows the efficiency of transfer learning. For the sake of analysis, the CNN classifier was trained for 60 epochs even though the validation accuracy reached 99% at the 22nd epoch. This result confirmed that transfer learning is very effective in saving the training time, while the issue of overfitting does not arise.

## 4. Skills for Increased Detectability

### 4.1. Detailed Categorization for Accurate Crack Detection

In previous literature related to crack detection, the deep learning model was trained for binary classes, e.g., Crack or Non-crack classes. Though the purpose of crack detection is detecting cracks against other objects, the binary classes do not result in accurate detection because of the presence of objects with similar visual patterns. Cracks have narrow linear shapes, while there are many other objects with similar, but not identical, shapes. In the case of binary classes, many objects with narrow linear shapes, such as joints, edges, corners, pipes, and electrical lines, will be detected as cracks. For example, Zhang et al. (2017) [32] reported that it is challenging to remove pavement edges in detecting asphalt cracks, or any other object which has a similar shape.

This research proposes a detailed categorization of the Non-crack class to overcome the limitation of crack detection techniques developed under idealized conditions. As stated in Section 2.3, five classes are used and the Crack class is only one of them. The Non-crack class is divided into four classes, two of which are Joint/Edge classes that include all possible linearly shaped objects.

**Figure 8:**
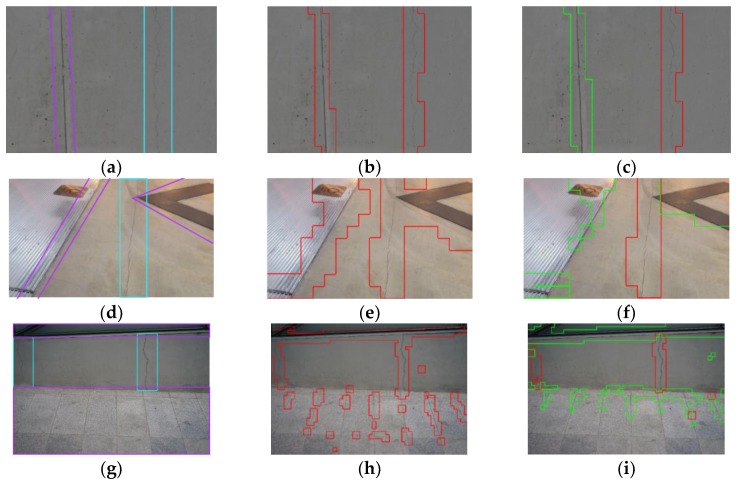
Examples to present enhancement by Joint/Edge Class: (**a**,**d**,**g**) original images, (**b**,**e**,**h**) classification results with two classes, Crack and Surface, and (**c**,**f**,**i**) enhanced classification results with introducing Joint/Edge(SL) class.

**Figure 9:**
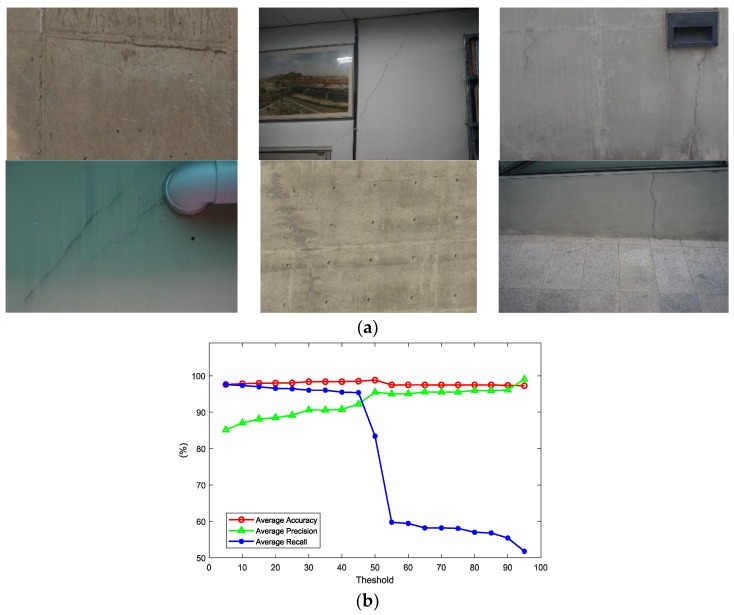
Parametric study of the probability threshold: (**a**) six crack images used for the parametric study; (**b**) performance measures of six images with increasing threshold.

Figure 8 illustrates the enhancement obtained by introducing multiple classes to consider the confusing linear-shaped objects. Figure 8a,d,g shows three examples that contains both cracks and confusing objects, which are a joint in Figure 8a, objects with linear edges in Figure 8d, and linear concrete edges and rectangular tiles in Figure 8g. In the images, cracks are marked with cyan boxes and the confusing objects are marked with purple boxes. Figure 8b,e,h is the classification results of three images when two classes, Cracks and Surfaces, are considered. As presented in red boxes, the confusing objects are misclassified as cracks, since their shapes are closer to cracks than surfaces. Figure 8c,f,i is the enhanced results when multiple classes are used. The highlighted part with green boxes shows the confusing objects are classified as the Joint/Edge (SL) class, and they have high agreement with the misclassified parts as cracks in Figure 9b,e,h. The average precision of the three images increased from 32.72% to 97.93% while the average recall slightly decreased from 100% to 98.93%. Thus, by separating the confusing objects from real cracks, the false positives can be minimized in the practical environments.

### 4.2. Parametric Study of the Probability Threshold

The threshold to determine cracks on the probability map may vary according to inspection purposes. In cases where precision is more important than recall, the threshold has to be relatively high, and vice versa. Mostly, recall is more valuable in the inspection of civil engineering structures, in order not to miss any possible source of failure. In this study, a parametric study to determine the proper threshold was conducted with six images obtained in diverse structural and photographing conditions as shown in Figure 9a. Three performance measures (e.g., accuracy, precision, and recall) were obtained by increasing the threshold on the six images as:

(2) $$\mathrm{Accuracy}=\frac{\mathrm{TP}+\mathrm{TN}}{\mathrm{TP}+\mathrm{TN}+\mathrm{FP}+\mathrm{FN}}$$

(3) $$\mathrm{Precision}=\frac{\mathrm{TP}}{\mathrm{TP}+\mathrm{FP}}$$

(4) $$\mathrm{Recall}=\frac{\mathrm{TP}}{\mathrm{TP}+\mathrm{FN}}$$

where TP is the true positive, TN is the true negative, FP is the false positive, and FN is the false negative. Their averages of six images calculated from 5% threshold to 95% in an increasing unit of 5% are illustrated in Figure 9b. The average accuracy changed only slightly in relation to the threshold, while the other two measures changed significantly. The average precision slowly increases from 0% to 50% and remains relatively constant afterwards. However, the average recall decreases little from 0% to 50% and drops suddenly after 50%. The significant change of both precision and recall around 50% results from the sliding window that scans all the pixels of an image twice. This result seems reasonable considering the case where a crack is at the center of a sliding window and at the border of the overlapped window. At first scanning, the pixels containing cracks would get high probability near 100%, while the crack may be missed at the second overlapped scanning. Though the result suggests the use of 50% as the threshold, the threshold is determined in order to make both precision and recall exceed 90% and to minimize the possibility of missing cracks with a small possibility of false positives. In the further detection using the probability map, the threshold is determined as 35% where precision starts exceeding 90% as show in the Figure 9b. The threshold is set to 35% to maximize recall value but users might change the threshold according to their purpose of inspection. 

## 5. Automated Crack Detection on Real Concrete Structures

### 5.1. Automated Crack Detection on Still Images

**Figure 10:**
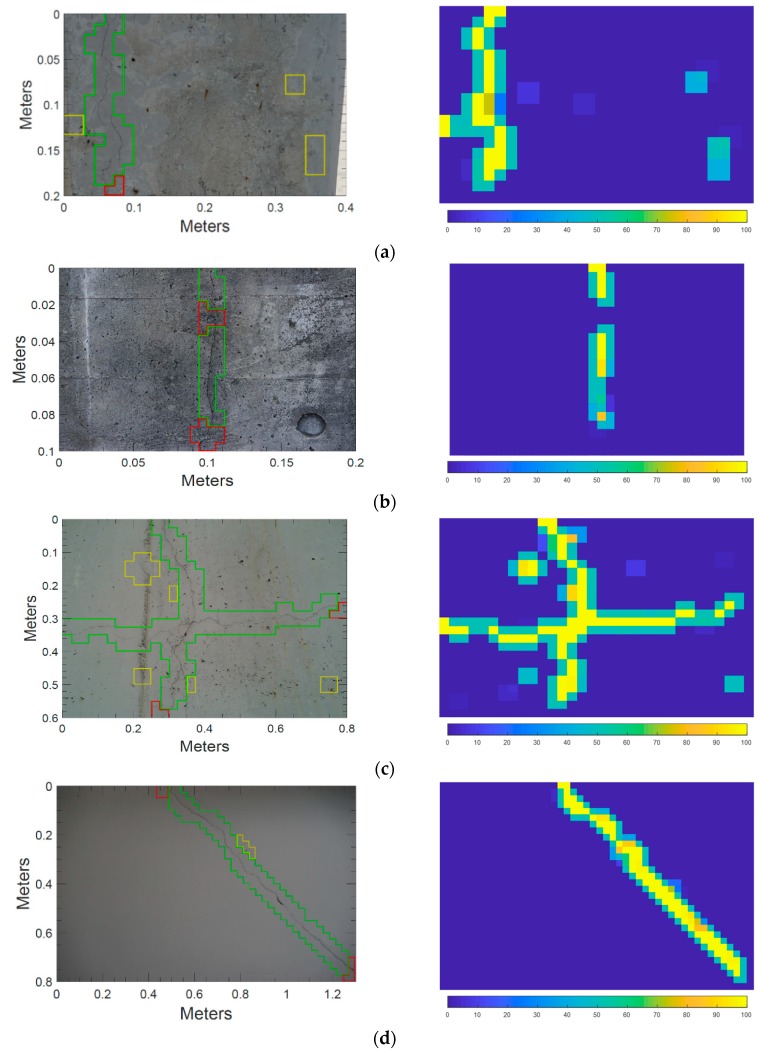

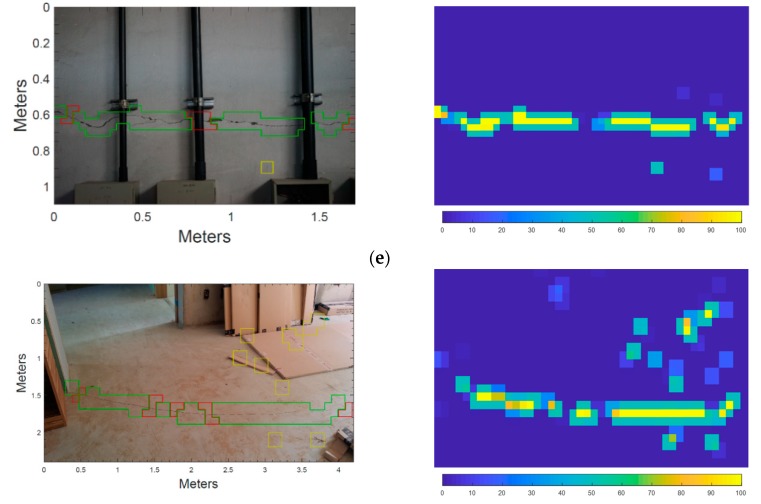
Crack detection result of each case by the proposed method (left column) and corresponding probability map (right column): (**a**) peeled concrete surface; (**b**) clean concrete surface; (**c**) concrete surface with many pores and construction joints; (**d**) dark concrete surface; (**e**) concrete surface with pipes and electric distribution boxes; and (**f**) floor with construction materials.

To validate the automated crack detection method, i.e., the applicability of the trained classifier to an on-site environment, tests were conducted with images taken from actual concrete structures with commercial DSLRs and smartphone cameras. The results of crack detection in Figure 10 show the performance of the proposed approach in extracting crack regions on concrete surfaces and the probability map corresponding to each image. In Figure 10, the distributions of true-positive (TP), false-negative (FN), and false-positive (FP) regions are highlighted as green, red, and yellow colored boxes, respectively. Remaining regions without highlight are true-negative (TN). The presence of cracks was detected successfully in all test images, though there were many obstacles. The obstacles in Figure 10a–d are stain, scratch, tie holes, and imprint of concrete mold that can be found on structure surfaces caused by poor handling and maintenance. Those in Figure 10e,f are pipes, electrical distribution boxes, and interior materials having a rectangular shape for the sake of convenience in construction. Despite the obstacles to crack detection varying according to each experimental environment, the proposed method successfully detected cracks as shown in Figure 10. Looking at the performance measures, the proposed method achieved more than 90% of accuracy for all the test images and attained an average precision of 86.73% and an average recall of 88.68% on the pixel-level, Table 2. The performance of the trained network was also tested on other 34 images of concrete surfaces which have similar patterns or textures and the results are tabulated in the Appendix A. The average accuracy is 97.02%, the average precision is 92.36% and the average recall is 89.28% for the result in Appendix A. 

**Figure 11:**
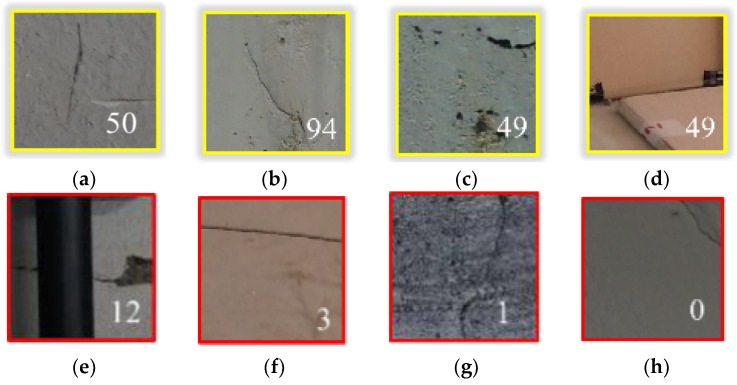
Example images of FP groups (**a**–**d**), FN groups (**e**–**h**) and their crack probabilities (**a**–**d**): (**a**) crack-shaped contaminants; (**b**) overlaid cement paste; (**c**) continuously distributed concrete pores; and (**d**) edge of linear-shaped construction material; (**e**) crack hidden behind object; (**f**) crack having straight line; (**g**) crack obscured by dark surface; and (**h**) crack on the corner of detecting window.

Despite of the excellent performance of the proposed method, it still has limitations in detecting cracks against objects that are indistinguishable in vision. Looking into the details, FPs were observed in various patterns that can be categorized into four groups. Figure 11a–d shows example images of four FP groups with their crack probabilities. The first group represents crack-shaped contaminants left on the surface as exemplified in Figure 11a; the second group is overlaid cement paste, as seen in Figure 11b; and the third group consists of continuously-distributed concrete pores, shown in Figure 11c. Due to their shapes, the possibilities of obtaining crack FPs for these three groups are estimated at over 35% (i.e., the threshold determined in Section 4.2). Under visual inspection, these FPs can be easily distinguished from cracks by checking for the existence of splits, which cannot be investigated in the monocular images. Instead, in order to reduce FPs in the proposed method, sufficient illumination may be used to unshadow these objects and contrast the split cracks; other techniques, such as stereovision [33] and infra-red, may also be implemented. The last group consists of a small number of discontinuous edges of linear-shaped construction material which are misclassified as cracks, Figure 11d. Though the proposed method suggested detailed categorization of surfaces to remove these patterns, a few which have relatively irregular shapes are classified as cracks. This FPs may be removed out by considering the region areas, shapes, and continuities in the further study. FNs are also observed in various patterns that can be categorized into four groups. Figure 11e–h shows the example images of four FN groups with their crack probabilities estimated as being low. The first group are the cracks hidden behind other objects as exemplified in Figure 11e. In this case, the major object in the window (e.g., the pipe) reduced the crack possibility while increasing the possibility of other classes (e.g., Joint/Edge (ML) and (SL)). The second group are the cracks having a linear shape without irregular patterns, as seen in Figure 11f. The training image categorization of the proposed method is based on the assumption that cracks generally have an irregular linear shape, and thus the second group is mostly classified into Joint/Edge (SL). These FNs are the result of inevitable trade-off in the process removing crack-like objects, and may be removed out by considering the region areas, shapes, and continuities. The third group are the cracks obscured by dark surfaces, shown in Figure 11g. This may be overcome by implementing sufficient illumination. The last group are the cracks located on the corner and boundaries of the detecting window, as in Figure 11h. Since this FN is related to the FOV, it may be solved by taking another image with an altered FOV or by using a video stream.

Based on the investigations of FPs and FNs, the four types of solutions suggested for reducing FPs and FNs are summarized in Table 3. This table shows that the proposed method can perfume excellently in practice if the solutions are combined in the further study.

### 5.2. Automated Crack Detection on Video Taken by Drone

There has been an increasing number of research on bridge inspection using unmanned aerial vehicles (UAVs) in the last several years because of their advantages such as safety and high productivity [34,35,36,37]. In this section, the feasibility of the proposed method is evaluated for UAV-based concrete structure inspection over still images.

**Figure 12:**
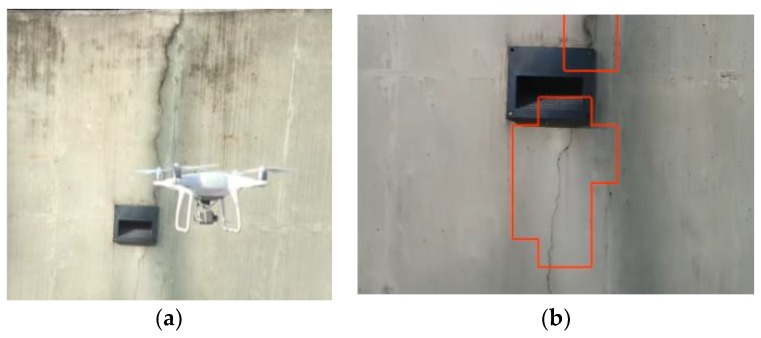
Automated crack detection using UAV: (**a**) Video shooting using UAV, (**b**) Example image of real-time crack detection.

The test video was taken at a concrete retaining wall located at the University of Seoul as shown in Figure 12. The wall has a varying height between 2 and 4 m, and a width of approximately 20 m. A region of 2 × 5 m was inspected using a drone, and 16 cracks were found visually with varying shapes and sizes. The wall is highly contaminated with sediments by leakage, and it attaches several pieces of rectangular sidewalk lighting equipment that initiate the cracks as in Figure 12.

The drone used in this study is Phantom 4 advanced (SZ DJI Baiwang Technology Co., Ltd., Shenzhen, China) equipped with a 1-inch 20-megapixel CMOS camera along a FOV 94° 20 mm lens. While shooting the video, the drone kept a distance from the concrete wall of about 2 m during the entire flight, and the approximate FOV was 0.75 mm × 1.60 m based on camera specifications. The working distance of 2 m was found to be sufficiently close for detecting major and minor cracks that formed on the concrete surface. The trained classifier was used to detect cracks from an image taken from the video every 0.5 s.

The real-time detection is demonstrated in the video [Link: https://youtu.be/5sNbfEaRwkU]. As shown in the video, the developed method successfully detected 15 out of 16 cracks, missing only a very small crack whose width was approximately 0.05 mm. For all images used in the detection, the precision and recall in the pixel level were calculated as 88% and 81%, respectively. Considering that the video frames taken by the moving UAV are quite blurred, the result shows that the proposed method is at the edge of practice for a UAV-based structure inspection.

## 6. Conclusions

This paper proposed an automated crack detection method based on deep learning to detect cracks on a large set of images taken on real structures. The entire procedure of the proposed method consists of fine-tuning AlexNet with Internet-based training images and crack detecting based on the probability map. The training and validation images covering diverse environments of on-site concrete structures were collected from the Internet using a web scraper. Data augmentation skills such as rotation, blurring, and color adjustment were implemented to enhance the diversity and quantity of the thousands of training and validation images. AlexNet was fine-tuned for five categories, Crack, Joint/Edge (ML), Joint/Edge (SL), Intact Surface, and Plant on 42,000 augmented images with 227 × 227 pixel resolutions. The probability map is suggested to strengthen the robustness of the sliding windows detection method. To construct the probability map, pixels accumulate the softmax layer value of the Crack class regardless of the highest detection result during the specified number of detections. After scanning all pixels, pixels with a larger value than a specified threshold are determined as cracks.

The comparative and parametric study examined the two main skills for increasing detectability of the CNN classifier, detailed categorization of the concrete surfaces and probability map. The comparative study was conducted to confirm how five categories increase the adoptability of the proposed CNN classifier for on-site inspection. The proposed CNN classifier was compared with a crack detection method based on a subtraction process and another CNN classifier having two classes: crack and intact surface. The proposed CNN classifier succeeded to extract the crack parts out of intact surfaces and objects having similar patterns to cracks while other methods determined the objects as cracks. An optimized threshold for the probability map was chosen through the parametric study. According to the result of the parametric study, the threshold is determined as 35% where both precision and recall are higher than 90%.

The performance of the proposed detection method using a CNN classifier was evaluated on 40 images representing on-site environments and a real-time video of a concrete wall taken using a UAV. Despite the existence of the concrete mold marks, pipes, and tie holes around cracks, the proposed CNN classifier successfully detected cracks against intact surfaces and similar objects. The average precision and recall for the images were 92.35% and 89.28% on the pixel level, respectively. The CNN classifier also succeeded in detecting cracks from the real-time video taken by the UAV at 3 frames per s with a recall of 81% recall and precision of 88%. The evaluation of results confirmed the applicability of the proposed method to on-site crack inspection.

This research confirmed that the CNN-based method shows a high degree of applicability for crack detection when proper skills are applied. The feature-extracting capability of CNN carries a huge advantage for computer vision-based inspection in civil and infrastructural engineering. The proposed method is beneficial in analyzing the crack morphology by eliminating the interrupting objects in an image in prior. Thus, the CNN-based method is expected to replace current visual inspection in the near future because of its automated feature-extracting capabilities and objective assessment of cracks.

## Figures and Tables

**Table 1:** Number of valid images scraped from the Internet.

Class	Keywords	Valid Images/Total Images
Crack	concrete crack	497/723
	concrete wall crack	573/703
	crack on concrete	537/683
	crack on concrete brick	429/905
	cement crack	485/681
	After Deleting Duplicates	2073
Joint/Edge	concrete corner	456/697
	concrete joint	225/794
	concrete tile	396/701
	grey concrete tile	446/705
	After Deleting Duplicates	1400
Plant	moss on concrete	654/757
	moss on concrete wall	773/929
	plant on concrete	452/890
	After Deleting Duplicates	1511
Intact Surface	cement texture	547/606
	concrete surface	518/853
	concrete texture	476/489
	concrete wall	489/644
	smooth concrete wall	493/619
	After Deleting Duplicates	2211

**Table 2:** Crack detection result of six images in Figure 10.

Image.	Resolution	Elapsed Time (s)	Accuracy (%)	Precision (%)	Recall (%)
(a)	3343 × 2191	1.63	96.25	93.67	94.22
(b)	4099 × 2773	2.43	97.46	100.00	71.19
(c)	4160 × 3120	2.85	96.09	86.72	97.32
(d)	5941 × 3961	4.26	99.03	94.33	95.87
(e)	6000 × 4000	5.31	98.5	94.74	86.86
(f)	4128 × 2322	2.88	92.53	50.93	86.67
Average		3.22	96.64	86.73	88.68

**Table 3:** Grouping of FPs and FNs, and their possible solutions.

False-Positive (FP)		False-Negative (FN)	
Groups	Solutions	Groups	Solutions
crack-shaped contaminants	1, 3	crack hidden behind object	4
overlaid cement paste	3	crack having straight line	2
continuously-distributed concrete pores	2	crack obscured by dark surface	1
edge of linear-shaped construction material	2	crack on the corner of detecting window	4

Solution 1: Enough illumination; Solution 2: Consideration of region areas, shapes, and continuities; Solution 3: Additional vision techniques (stereovision, IR, etc.); Solution 4: Moving image FOVs (e.g., using a video stream).

## Appendix A

**Table A1:** Crack detection result of forty images.

Image No.	Resolution	Elapsed Time (s)	Accuracy (%)	Precision (%)	Recall (%)
1	3343 × 2191	1.63	96.25	93.67	94.22
2	4099 × 2773	2.43	97.46	100.00	71.19
3	4160 × 3120	2.85	96.09	86.72	97.32
4	5941 × 3961	4.26	99.03	94.33	95.87
5	6000 × 4000	5.31	98.50	94.74	86.86
6	4128 × 2322	2.88	92.53	50.93	86.67
7	5875 × 3943	4.59	98.77	86.85	96.96
8	5101 × 3805	4.30	98.50	100.00	92.19
9	2515 × 2101	1.09	95.33	100.00	63.42
10	2431 × 2047	0.94	97.82	100.00	90.26
11	1107 × 925	0.39	98.44	100.00	93.66
12	5863 × 3877	4.74	97.51	90.22	79.05
13	3953 × 2593	2.20	94.85	87.19	80.14
14	1960 × 1540	1.11	96.94	94.86	100.00
15	3656 × 3082	2.14	98.48	97.20	96.27
16	5496 × 3670	4.50	100.00	100.00	100.00
17	2425 × 2095	1.06	96.27	100.00	86.68
18	6000 × 4000	4.84	97.92	80.11	95.02
19	3421 × 1987	2.59	95.99	94.60	95.92
20	1855 × 1153	0.98	98.09	90.78	98.84
21	1969 × 1369	0.93	93.76	90.40	67.17
22	1052 × 1000	0.60	98.96	100.00	95.37
23	4160 × 3120	2.60	97.92	93.75	83.82
24	2119 × 1411	0.94	96.40	95.12	94.62
25	1481 × 947	0.71	92.13	100.00	83.24
26	1442 × 926	0.57	90.04	100.00	48.15
27	1742 × 930	0.71	100.00	100.00	100.00
28	1506 × 931	0.55	94.61	55.81	100.00
29	1064 × 732	0.42	98.61	100.00	93.81
30	4096 × 2160	1.70	99.38	100.00	97.27
31	819 × 614	0.49	98.15	100.00	92.23
32	4160 × 3120	3.36	98.44	94.48	94.21
33	4597 × 3175	3.55	95.67	61.91	89.77
34	1456 × 937	0.58	98.34	90.48	100.00
35	3120 × 4160	3.0	98.98	91.13	96.86
36	3094 × 2174	1.91	95.73	100.00	57.25
37	1891 × 925	0.88	100.00	100.00	100.00
38	1723 × 914	0.65	96.54	95.76	86.65
39	1480 × 935	0.68	97.10	97.06	90.32
40	1828 × 939	1.39	95.17	86.12	100.00
Average			97.02	92.36	89.28
